# Overview of the Evolution of Silica-Based Chromo-Fluorogenic Nanosensors

**DOI:** 10.3390/s19235138

**Published:** 2019-11-23

**Authors:** Luis Pla, Beatriz Lozano-Torres, Ramón Martínez-Máñez, Félix Sancenón, Jose V. Ros-Lis

**Affiliations:** 1 Instituto Interuniversitario de Investigación de Reconocimiento Molecular y Desarrollo Tecnológico (IDM), Universitat Politècnica de València, Universitat de València, Camino de Vera s/n, 46022 Valencia, Spain; plablas@upv.es (L.P.); bealotor@upv.es (B.L.-T.); fsanceno@upv.es (F.S.); 2 Unidad Mixta de Investigación en Nanomedicina y Sensores, Universitat Politècnica de València, IIS La Fe, Valencia, Spain; 3 Unidad Mixta UPV-CIPF de Investigación en Mecanismos de Enfermedades y Nanomedicina, Valencia, Universitat Politècnica de València, Centro de Investigación Príncipe Felipe, València, Spain; 4 Departamento de Química Inorgánica, Universitat de València, Doctor Moliner 56, 46100 Valencia, Spain

**Keywords:** nanoparticles, silica, mesoporous, color, fluorescence, sensor, molecular recognition, arrays, gated materials

## Abstract

This review includes examples of silica-based, chromo-fluorogenic nanosensors with the aim of illustrating the evolution of the discipline in recent decades through relevant research developed in our group. Examples have been grouped according to the sensing strategies. A clear evolution from simply functionalized materials to new protocols involving molecular gates and the use of highly selective biomolecules such as antibodies and oligonucleotides is reported. Some final examples related to the evolution of chromogenic arrays and the possible use of nanoparticles to communicate with other nanoparticles or cells are also included. A total of 64 articles have been summarized, highlighting different sensing mechanisms.

## 1. Introduction

The development of sensors, in particular, chemical sensors, has been an area of wide interest in recent decades [1]. Sensors can transform chemical information in the environment into a macroscopic signal that can be analyzed. From the range of sensors available, optical probes offer extraordinary potential. They are usually cheap and versatile, and some of them can be easily printed on surfaces. In particular, optical probes based on changes in color can be used without expensive instrumentation, as the signal can be easily detected by the naked eye. Besides, color modulations can be followed using smartphones, cameras, or other image capturing systems. Few technologies are as advanced or as inexpensive as visual imaging [2,3,4]. Traditionally, the most common approaches to design optical probes have been: (i) linking a chromophore with a receptor unit by means of a covalent bond, (ii) the use of competition assays between a dye bonded to a receptor and a certain analyte, and (iii) the use of new molecular systems that undergo analyte-induced chemical reactions coupled to suitable colorimetric events. 

Moreover, the incorporation of nanomaterials in optical probes has resulted in novel properties and new sensing paradigms to be explored. At its most basic level, nanomaterials can act simply as supports in which a probe is attached or adsorbed. In a second level of sophistication, nanomaterials can participate actively in the sensing mechanism and provide improved selectivity or sensitivity in combination with the optical probe [5,6].

**Figure 1:**
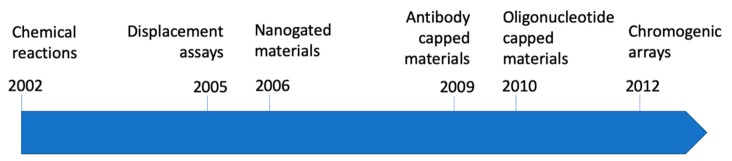
Timeline of the first examples developed by the authors in the different categories of the review.

Among nanomaterials that can be used for the development of optical chromo-fluorogenic probes, silica-based supports offer several advantages, including the possibility of (i) having mesoporosity, (ii) control of particle size, and (iii) easy functionalization of the surface with a wide range of organic and inorganic molecules. Moreover, silica-based materials are transparent to the light in the visible zone [7]. The evolution of silica-based chromo-fluorogenic probes has occurred mainly in the last 20 years. Advances have been consequence of the combination of diverse technologies that merged to offer new protocols in sensing systems. The combination of mesoporous silica with gatekeepers, concepts such as binding pockets, data analysis, etc., allowed the development of new approaches that improved sensitivity, resulting in the development of new sensing paradigms. The authors have been deeply involved in the topic during such period, and the evolution of its research has gone in parallel to the general evolution of the discipline. As can be seen in Figure 1, studies went from organic dyes attached to silica surfaces to more sophisticated nanogated materials with an increasing presence of biomolecules as sensing systems. A parallel development during these years has been the integration of different optical probes in chromo-fluorogenic arrays. The present review aims to describe, through relevant research developed in our group, an overview of the evolution of the advances in the area of silica-based chromo-fluorogenic probes during the 21st century.

## 2. Systems Based on Dyes and Reactive Units Grafted onto Surfaces 

The first example developed by us using silica-based nanomaterials as the active agent was based on the tendency of fluoride to attack silica in acidic media. This property was exploited for the development of a chromo-fluorogenic probe for fluoride [8]. In this research, MCM-41 was chosen as a mesoporous silica support and covalently functionalized with three dyes (9-anthraldehyde, 4-{2-[4-(dimethylamino)phenyl]diazenyl} benzoic acid and lissamine rhodamine B sulfonyl chloride). Solutions of acetonitrile:water 7:3 *v*/*v* at pH = 2.5 containing the functionalized silica particles became colored in the presence of fluoride due to fluoride-induced disintegration of the silica support that resulted in the release of the dyes to the solution. A linear response for the dye released versus the concentration of fluoride was observed. The material was tested for the analysis of fluoride in commercial toothpaste with good results.

**Figure 2:**
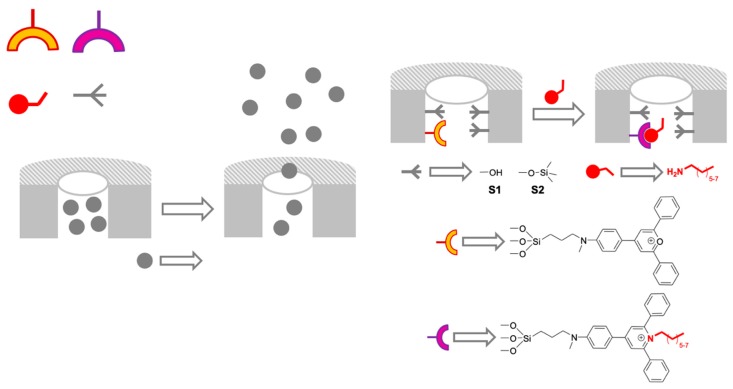
Scheme of the mesoporous silica materials S1 and S2 functionalized with pyrylium moieties anchored in the inner surface of the pores for the detection of medium chain primary amines.

In contrast to the previous example, in which the analyte reacts with the support, in most sensing systems, the analyte reacts with molecules attached to the surface. In the particular case of using porous materials, sensing materials can additionally benefit from the “binding pockets” concept, in which the mesopores of mesoporous silica materials are functionalized and act as pockets able to coordinate selected guests. The use of binding pockets to modulate the selectivities of chemical reactions is widely used in biological systems; in particular, in proteins and enzymes. For instance, enzymes hide active centers inside their structures, which are accessed by channels. Only those molecules that simultaneously fulfill the requirements of being able to both, access the active center, and suffer a specific reaction/coordination, will react. In the case of the materials, the protein structure is substituted by the porous structure (see Figure 2). 

A first example of the use of binding pockets is based on the reaction of pyrylium derivatives with amines to give pyridinium salts (Figure 2). Three different solids based in silica nanoparticles were prepared for the chromogenic discrimination of primary aliphatic amines in water [9]: (i) a mesoporous silica material functionalized with the pyrylium cation (S1), (ii) a mesoporous silica material functionalized with the pyrylium cation and trimethylsilyl groups (S2), and (iii) a non-mesoporous silica material functionalized with the pyrylium cation (S3). All solids were exposed to amines with different chain lengths. The most remarkable result was the high level of selectivity displayed by S2. It shows a selective color shift associated with the formation of the pyridinium salt only for the “medium”-chain amines (C_7_ to C_9_), whereas amines with longer or shorter chains did not display any significant color change. Furthermore, the presence of the dye inside the pores offered protection against other substances, such as ions usually present in water, alcohols, thiols, and secondary and tertiary amines that could also react with the pyrylium cation. S1 remained unreacted with amines, probably due to its hydrophilic character that challenges the diffusion of amines from water to the pyrylium groups in the pores. The nonporous solid S3 reacted with the medium and long chain amines but it was not selectivity found to react with S2. The remarkable enhanced selectivity of S2 in front of the two other solids can be explained by a combination of the hydrophobicity of the surface and the porous system. Whereas the hydrophobic surface favors the extraction of the medium and long chain amines, the diffusion of the pores is easier for shorter amines, the final colorimetric behavior being a compromise between these two factors.

The concept of binding pocket was also applied for the sensitive detection of biogenic amines [10]. As noted above, pyrylium compounds react unselectively with amines to give the corresponding pyridinium derivatives, with dramatic color changes from blue to red and an enhancement of fluorescence emission. In this case, a phenyl vinyl pyrylium derivate was included in three different supports: (i) a hydrophobically functionalized disordered mesoporous silica (S4), (ii) non-porous hydrophobic silica (S5), and (iii) a PVC membrane (S6). Suspensions of the materials in water (pH = 10.5) showed clear spectroscopic changes associated with the formation of the pyridinium compounds only for biogenic amines (histamine, putrescine, and cadaverine), whereas no color change was observed in the presence of amino acids, even at high concentrations. The discrimination of the biogenic amines in front of amino acids is especially remarkable for amino acids with nucleophilic residues, such as histidine, lysine, and cysteine. Selectivity can be assigned to the confinement of the dye inside the hydrophobic pores that protect them from the nucleophilic attack of the charged species (amino acids) but allow the diffusion of the neutral ones (biogenic amines). Although the behavior of the three sensory materials was similar, the kinetics of the reaction wereconditioned as a function of the pore size and the lipophilicity of the cavity. Finally, the ability of the mesoporous silica material for the detection of biogenic amines was tested in extracts of fish (*Sparus aurata*) spiked with increasing amounts of histamine. Gradual colorimetric changes were observed by the naked eye, from blue to red-orange as a function of histamine concentration. 

Long chain carboxylates were selectively detected in aqueous environments using the biomimetic binding pocket approach in combination with a urea-containing fluorescent dye [11]. The ability of the urea and thiourea groups to coordinate carboxylate has been widely explored for sensing purposes. Nevertheless, in most cases, the interaction is observed only in organic solvents due to the competition of the urea with the hydrogen bonds formed by water molecules in aqueous environments. To solve such inconvenience and gain selectivity, a 7-amino-phenoxazin-3-one derivative was grafted onto the inner of the pores of the mesoporous support through a urea bond and the resulting material hydrophobized with hexamethyldisilazane. The sensory material was able to selectively extract from aqueous environments, long chain carboxylates (such as decanoate, dodecanoate, tetradecanoate, hexadecanoate, octadecanoate, oleate, and linoleate) over short chain carboxylates (acetate, butanoate, hexanoate, and octanoate), small cations and anions, and biological molecules commonly present in plasma. Again, this selectivity can be assigned to the hydrophobic character of the inner pores of the sensing material. Once inside the pores, long chain carboxylates interacted with the urea biding site unit, inducing marked color changes and emission enhancements of the phenoxazin-3-one signaling unit. 

A hybrid material with a dual role (detection and adsorption) toward the Hg(II) cation was prepared using a mesoporous material as an inorganic scaffold [12]. It explores the strong affinity of mercury for sulfur containing compounds. For the preparation of the final sensing material the surface of the thiol modified silica was allowed to react with a blue squaraine dye forming the colorless adduct 2,4-bis(4-dialkylaminophenyl)-3-hydroxy-4-alkylsulfanylcyclobut-2-enone compound grafted onto the surface. Hg(II) is able to react with the sulfur atoms of the adduct recovering the structure of the fluorescent and deeply colored squaraine that is released to the solution. The probe offered a large dynamic concentration range and a limit of detection as low as 0.1 ppm. Other metal cations (Cu^2+^, Fe^3+^, Zn^2+^, Ni^2+^, alkaline and alkaline earth) and anions ubiquitously present in water induced no release of the dye. Only other thiophilic cations such as Pb^2+^ or Cd^2+^ lead to very minor release. Besides, the prepared material was also able to adsorb Hg(II) with high efficiency and the solid can be reused upon Hg(II) elimination from the solid at acidic pH. Again, the mesoporous structure not only acts as innocent support but has an active role in the metal sensing and removal processes. Analogous materials were prepared with silica gel and silica fumed instead of mesoporous silica did not offer the release of the dye, even at 1000 ppm. It seems that the concave geometry of the pores favors the reaction of the metal with the adduct moieties in the confined space, and this results in a more efficient release of the squaraine dye.

Other hybrid materials functionalized with reactive units for the detection of nerve agents (sarin, tabum, and soman) [13] and sulfite [14] were also described.

As an evolution of the binding pocket concept, the access to the reaction site can be controlled, apart from through the control of the diffusion carried out by the pores, by steric hindrance on flat surfaces. Based on the above-mentioned facts, the formation of the colorless thiol-squaraine adduct has also been the basis of a series of colorimetric sensors based in the control of accessibility of a squaraine dye (D) from the solution to the surface of a functionalized material. For instance, a pyrophosphate colorimetric probe was developed using fumed silica nanoparticles covalently functionalized with thiol groups as reactive units (R) and a linear polyamine as a host site (H) [15]. The sensing paradigm relies on the bleaching of the squaraine dye, after reaction with the thiol moieties in the surface of the solid, which was modulated in the presence of pyrophosphate via the formation of complexes between this anion and protonated polyamines (H). The formation of above-mentioned complex controls the accessibility and reactivity of squaraine dye with R. In the absence of pyrophosphate, the reaction between D and R turns the solution colorless, whereas in the presence of pyrophosphate, this anion forms complexes with H inhibiting the reaction between D and R, so that the solution remains blue. The system was assayed with phosphate, sulfate, nitrate, chloride, and perchlorate as well. The best selectivity and sensitivity for pyrophosphate anion was found at pH 5 with a negligible responses for the other anions tested.

**Figure 3:**
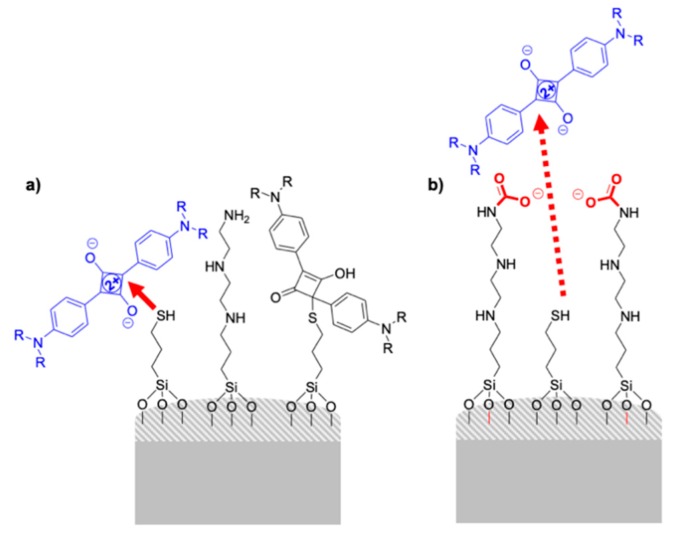
Scheme of the silica-based material functionalized with thiols and amines for the detection of CO_2_ through control of mass transfer of the dye from the solution to the material surface. In the absence of CO_2_ (**a**) squaraine dye can access to the surface and react with the grafted thiols. However, in the presence of CO_2_ (**b**) squaraine-thiol reaction is inhibited.

Another sensing application, also using polyamine and thiol functionalized mesoporous silica nanoparticles (MSNs), was reported for the detection of carbon dioxide (CO_2_) [16]. The carbon dioxide reacts with the amines covering the particles with bulky carbamate groups that avoid the access of the squaraine dye to the thiols on the surface of the silica material (Figure 3). ^1^H-NMR experiments in D_2_O confirmed the formation of carbamate groups. Experiments were carried out in neutral PBS, and selectivity was successfully demonstrated as the probe remained silent in the presence of other gases such as CO, CH_4_, NH_3_, NO_X_, and SO_2_ and acidic vapors. The possible use of the material as a dosimeter for CO_2_ was tested exposing the probe to an atmosphere containing different CO_2_ and N_2_ mixtures. A limit of detection (LOD) of 2.19% CO_2_ was determined. 

A similar strategy was explored for the colorimetric detection of formaldehyde [17]. Nanoparticles covered with thiol and polyamine groups were prepared and tested against formaldehyde and other aldehydes. Formaldehyde reacts with the thiol groups to form an alkythio-methanol. It transforms the surface coating groups from reactive thiols to unreactive alcohols that are not able to induce bleaching in the squaraine. The material shows remarkable selectivity towards formaldehyde in front of other compounds containing carbonyl groups, such as acetaldehyde, propionaldehyde, butyraldehyde, acetone, salicylaldehyde, and 4-aminobenzoic acid. This selectivity is attributed to the polyamine units that generate a highly polar environment around the thiols that hinders the access of other molecules; only formaldehyde, the more electrophilic and smallest of all the molecules tested, was detected. This hypothesis was validated with the preparation of one material grafted with thiols but without polyamines, with a significant reduction of the selectivity. A LOD of 1.2 µM (36 ppb) was obtained for formaldehyde.

Using the same approach, materials functionalized with binding sites and reactive units for the sensing of heparin [18], nerve agent mimics [19], and anionic surfactants [20] were described.

**Figure 4:**
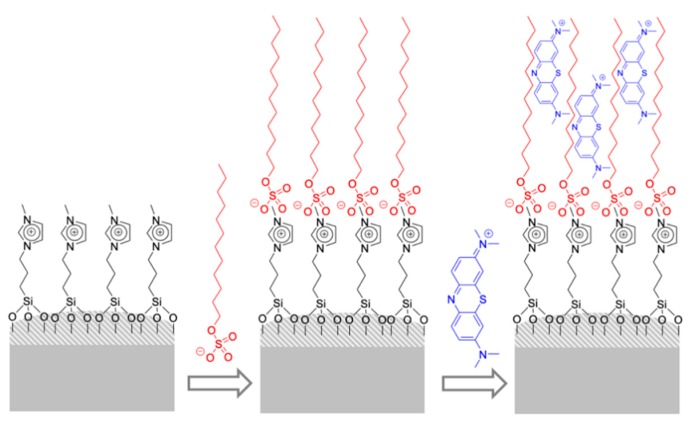
The scheme of a two-step protocol for the detection of anionic surfactants (SDS) with a silica inorganic support functionalized with imidazolium cations (acting as binding sites) and treatment with methylene blue.

Flat silica was also used for the design of a hybrid system functionalized with a trialcoxisilane–imidazolium salt derivative for the detection of anionic surfactants [21]. The recognition mechanism involved two steps. In the first step, the anionic surfactant interacts with imidazolium cation, resulting in the formation of a strong hydrophobic monolayer. Then, in a second step, the monolayer can extract an adequate dye, such as methylene blue, from the water solution, resulting in the bleaching of the solution, whereas the solid gets colored (Figure 4). Color changes were visible to the naked eye. In a typical experiment, the sensing material was suspended in a lauryl sulfate aqueous solution, then the solid was filtered and suspended in a methylene blue solution. The final solution was colorless, whereas the solid became deep blue. Besides, the response of the material as a function of lauryl sulfate concentration was studied by measuring the absorbance at 664 nm due to the methylene blue remaining in solution after the two-step protocol mentioned above and a clear color modulation was observed by the naked eye (LOD = 1 ppm). A similar response was observed for tetradecyl sulfate, alkylbenzene sulfonate, and partially with dodecyl phosphate. Possible interferents, such as anions (carbonate, phosphate, sulfate, nitrate, and chloride), cations (Na^+^, K^+^, Ca^2+^, and Mg^2+^), neutral (Triton X-100), and cationic surfactants (CTAB, cetyltrimethylammonium bromide) did not produce any response. The dependency of the response on the chain length was also studied through the reaction of the solid with different alkyl sulfates. The solid responded to linear alkyl sulfates with 14 and 12 carbons and partly to decylsulfate, whereas for octylsulfate and hexylsulfate no response was observed. The material was validated by the determination of anionic surfactants in residual waters.

Spiropyrans are cyclic neutral molecules capable of being transformed into charged, linear merocyanines with a positively charged nitrogen atom in each structure. Both structures coexist in equilibrium as a bistable system and can be reversibly transformed one into the other depending on the external conditions, such as light, pH, or temperature. The interconversion of merocyanine to spiropyran controlled by polarity changes was explored for the detection of long-chain carboxylates [22]. For this purpose, a spirobenzopyran derivative was anchored to the surface of mesoporous silica nanoparticles, together with a *N*-phenyl-*N*′-propylthiourea, which was employed as an anion binding site. The resulting material starts with the cationic merocyanine and shows an intense pink color in water. The material was tested against a family of carboxylates with different chain lengths (from acetate to docecanoate), showing that the color changed as a function of the chain length. The proposed mechanism is the formation of a monolayer on the material due to the coordination of the carboxylic acids to thiourea groups present in the surface of the material. This decreases the polarity in the environment of the dye, inducing the conversion from the pink merocyanine to the pale yellow cyclic neutral structure. The system shows selectivity towards carboxylic acids in front of lauryl sulfate, another anionic surfactant. Also, nanoparticles functionalized only with the spiropyran derivative were prepared for the elucidation of the role played by the binding site. These particles did not show any response, demonstrating the relevance of the interaction between the thiourea and the carboxylic groups in the sensing protocol. 

Silica-based colorimetric nanosensors can be incorporated into polymers and textiles, benefiting from the combination of nanomaterials with a macroscopic support. A novel naked eye sensing probe was developed for the detection of strong acids combining chemical pH indicators, textile fabrics, and mesoporous silica [23]. Silica nanoparticles were functionalized with 2,2′,4,4′,4″-pentamethoxy triphenyl methanol (pentamethoxy red pH indicator). The material was fixed to a cotton textile with a nitrocellulose polymer. Pentamethoxy red was selected because of its response to pH changes; it is colorless at neutral, turning to an intense reddish violet color in the presence of strong acids (pKa = 1.86, approximately pH 1). Covalent attachment of the dye to nanoparticles and resin immersion did not affect its indicator properties, but moved the turning point towards less acidic pH, making possible the detection of weaker acids (pH 2.5–3.5) and confirming that resin-encapsulation reinforced the stability of the dye. The cotton textile modified with the dye loaded silica was washed several times, keeping over 97%, the color intensity at pH 0. Besides, the reversibility of the textile was tested in solution and air, showing a high degree of reversibility when the acid was detected in solution but low when it was done on air. These results confirmed the potential of the system for its incorporation into personal protection equipment for reducing the incidence of acidic burns.

## 3. Systems Based on Displacement Assays

In the indicator displacement assays, the indicator coordinated with the receptor through electrostatic and supramolecular interactions forms a non-covalent complex. The sensing paradigm is based on the fact that the analyte presents a stronger binding constant than the indicator with the receptor, which results in a displacement of the indicator. The displacement of the indicator from the receptor is accompanied by differences in the optical properties of the indicator that are can be easily measured. The systems must be prepared taking into account that the constant between receptor and the interferents should be lower than those between the receptor and dye (K_interferent_ < K_dye_). Moreover, the displacement of the dye will occur only when the constant between the receptor and the dye is lower than that between the target analyte and the receptor (K_dye_ < K_analyte_). This approach appears as an alternative that requires less synthetic effort than traditional chemosensors, in which the signaling unit and the receptor unit are linked by covalent bonds. Although initially this approach was first developed for molecular chemosensors, we reported how this displacement protocol could also be applied when using certain materials.

**Figure 5:**
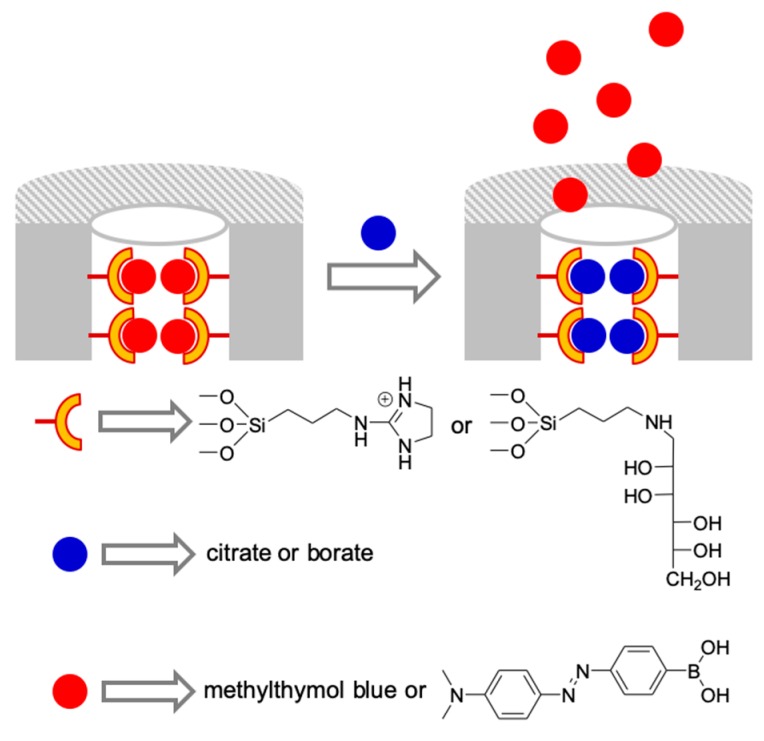
Mesoporous silica functionalized with binding sites and loaded with dyes for the selective detection of citrate and borate anions using a displacement assay.

The idea of the displacement assays together with that of the “binding pocket” was used for the selective sensing of anions (Figure 5). Two silica mesoporous materials were prepared for the sensing of citrate and borate [24]. In the material designed for the sensing of citrate, the silica was functionalized with guanidinium salts, a functional group able to undergo hydrogen bonding interactions with carboxylate groups, and loaded with the anionic dye methylthymol blue. Tats dye was selected because contains four carboxylate groups. The sensing material was suspended in water (ph = 7) in the presence of different carboxylates (citrate, succinate, lactate, malate, acetate, oxalate, propionate, formate, tartrate, maleate, malonate, glutarate, adipate, pimelate, and phtalate) and the absorbance band centered at ca. 604 nm, ascribed to methylthymol blue dye, was measured in the solution. A citrate selective response was observed. Again, the effect of the pores is critical; a material prepared with the same functionalization but using silica fumed as support, therefore, without mesopores, showed a very poor response in comparison with the mesoporous material. The same strategy was used for the recognition of borate. In this case, the mesoporous material was functionalized with mannose and loaded with an azoic dye containing one boronic acid which has a high affinity towards carbohydrates (Figure 5). When the material was suspended in water containing borate, the azoic dye was selectively displaced and was measured through its absorbance in the solution at ca. 455 nm.

In another example, the combination of the displacement assays approach together with the concept of binding pocket was employed for the detection of phosphate anions [25]. In this case, the mesoporous silica materials were functionalized with amines inside the pores and loaded with different carboxylate-containing dyes (Methyl Red, carboxyfluorescein, and Methylthymol Blue). These dyes interact with partially protonated amines inside the pores (at pH 7.5) via hydrogen bonding and electrostatic interactions. In the presence of anions, a polyammonium-anion complex is formed, with the subsequent displacement of the dye from the pores to the solution. Suspensions of the solids in water (HEPES, 4-(2-hydroxyethyl)-1-piperazineethanesulfonic acid, 0.01 M pH = 7.5) were exposed to fluoride, chloride, bromide, iodide, phosphate, sulfate, acetate, and nitrate anions for 10 min, and the absorbance at the corresponding solutions was measured. Phosphate induced a remarkable displacement of the dye from the pores to the solution. The addition of sulfate also induced some color but to a lesser extent than phosphate. The solutions remained colorless in the presence of the rest of anions. The displacement of the dye correlated well with the binding constants of the anions with polyamines. Moreover, the response to the anions was highly dependent on the nature of the indicator loaded in the pores. The highest absorbance in the solution in the presence of phosphate was observed for 5-carboxyfluroescein, a dianion. By contrast, Methylthymol Blue was poorly released in the presence of phosphate. This later dye is a trianion able to form too strong complexes with the poliamines and cannot be effectively displaced by phosphate. Finally, the role of the nanoscopic binding pockets was demonstrated by comparing the carboxyfluorescein-loaded mesoporous silica material with a similar silica gel support without pores. Suspensions of the later in the presence of above-mentioned anions did not show any response, which demonstrates that “binding pockets” favor the spatial proximity of amines allowing the preferential coordination-discoordination of the dye in absence or presence of phosphate anion.

The influence of the binding sites was further studied by the preparation of five mesoporous silica materials functionalized with different binding groups: (i) primary amine, (ii) guanidinium, (iii) urea, (iv) imidazolium, and (v) quaternary ammonium [26]. Some 5-carboxyfluorescein was loaded inside the pores. The solids were suspended in aqueous solutions (HEPES pH 7.5) containing carboxylate anions such as acetate, citrate, lactate, succinate, oxalate, tartrate, malate, mandelate, and glutamate and the absorbance of the dye released was measured in the solution. The response observed was in agreement with the relative binding constants of the analytes and the dye with the binding groups. For example, for the materials containing imidazolium and quaternary ammonium groups, the amount of dye displaced from the pore voids followed the order citrate > succinate~oxalate~tartrate~malate > acetate~lactate~mandelate~glutamate. This order was in agreement with the nature of the binding sites that could display only electrostatic interactions. On the contrary, the material containing urea binding units (a neutral unit with hydrogen bonding capacity) showed similar optical responses for all the carboxylates independent of their charge. The behavior of the guanidinium-functionalized solid was in agreement with its capability of forming both electrostatic and hydrogen bonding interactions. The better selectivity was found for the solid containing amines for citrate detection.

**Figure 6:**
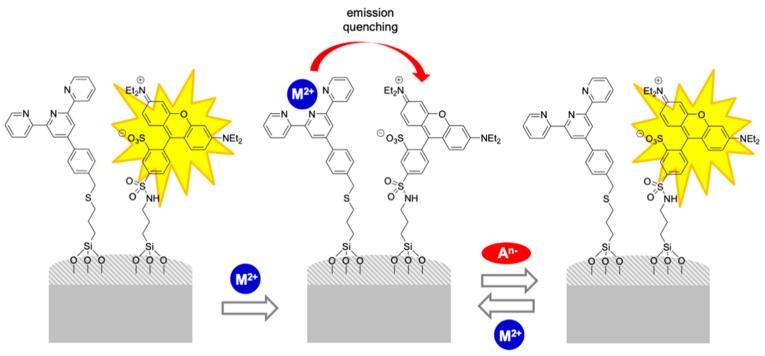
Silica nanoparticles functionalized with terpyridine (cation binding units) and sulforhodamine B (reporter) for the fluorescent recognition of anions.

Silica nanoparticles functionalized with sulforhodamine B as an indicator unit and terpyridine as a coordination/receptor site were prepared for the differential sensing of anions by using a quencher displacement assay (Figure 6) [27]. Terpyridines are not able to quench the sulforhodamine B fluorescence. However, in the presence of metal cations, colored M^2+^-terpyridine complexes are formed that switch off the material’s fluorescence. The addition of the target analyte, which shows a higher affinity for the metal than the receptor (terpyridine) displaces the metal cation from the receptor, leading to the revival of the fluorescence of the nanoparticles. The sensor can be regenerated simply by the addition of another cation, which recovers the quenching process. Energy transfer processes involved in the quenching events are dependent on the separation between the fluorophore and the quencher. Thus, the inclusion of both units simultaneously on the same surface allows an efficient quenching that reaches 99% of the rhodamine emission. Emission lifetime measurements indicate that it is a process dependent on the distance between the metal cation and the fluorophore and that the close distance between the receptor and the fluorophore obtained by functionalization in the same particle is a key issue. Different heavy cations, such as Fe^3+^, Hg^2+^, Cu^2+^, Ni^2+^, and Pb^2+^, were used inducing quenching, and different anions, such as H_2_PO_4_^−^, HSO_4_^−^, F^−^, Cl^−^, Br^−^, I^−^ and NO_3_^−^, were assayed. The materials loaded with Fe^3+^, Hg^2+^, Cu^2+^, or Ni^2+^ showed poor selectivity although, as a general trend, the addition of H_2_PO_4_^−^ or F^−^ offered stronger increments of the fluorescence intensity than the rest of anions. However, the hybrid material loaded with Pb^2+^ offered a selective response. Only the addition of H_2_PO_4_^−^ revived the sulforhodamine fluorescence, whereas the other anions remained largely passive. Using this procedure, concentrations as low as 5 ppm of H_2_PO_4_^−^ can be detected.

## 4. Chromogenic Arrays

Chemical sensors sometimes can offer limitations in complex systems such as food matrixes or body fluids. They are generally based on a single compound and have some limitations derived from a lack of specificity (offering false positives or false negatives). Thus, although some attempts have been made in single analyte indicators, a promising, potent, and versatile approach to be applied in complex matrixes is the use of chromogenic arrays. They consist of a combination of responsive dyes with a statistical analysis of the data. When these arrays are used in gaseous samples, they are known as optoelectronic noses [28]. 

Chicken meat aging was tested using an optoelectronic nose based on the use of 16 pigments (prepared after adsorption of selected dyes into UVM-7, silica, and alumina) [29]. The dyes included pH indicators (phenolphthalein, malachite green, *m*-cresol purple, phenol red, br-cresol purple, dimethyl yellow); hydrogen bonding derivatives (1-butyl-3-(4-nitro-phenylazo)-phenylthiourea); chemosensors which reacted with sulfur and amine functional groups (2,6-diphenyl-4-(2-(4-*N*,*N*-dimethylaminophenyl)vinyl), pyryliumtetrafluoroborate, and 4-(4-*N*,*N*-dimethylphenyl)-2,6-diphenylpyrylium perchlorate); Lewis acids (dinuclear rhodium complex); and several natural dyes (curcumin, carminic acid, litmus). The color of an array of the 16 pigments changed markedly with chicken ageing in a packaging atmosphere (70% nitrogen, 30% carbon dioxide). RGB color coordinates were used for the generation of difference maps which were analyzed using principal component analysis (PCA). This PCA analysis showed a marked dispersion with nine dimensions required to explain 95% of variance. Besides, a 3D representation of the three principal components was able to differentiate aging with a two-day interval. On the other hand, PLS analysis allowed the preparation of a model to correlate the chromogenic data from the array with chicken meat aging.

A similar idea of chromogenic arrays as optoelectronic noses was used to monitor boiled, marinated turkey freshness [30]. The array combined 13 different indicators (pH, nucleophilic sensing dyes, etc.) with three inorganic supports (UVM-7, alumina, and silica gel). As an example, reactants sensitive to sulfur and amine functional groups (i.e., 2,6-diphenyl-4-(2-(4-*N*,*N*-dimethylaminophenyl)vinyl)-pyrilium tetrafluoroborate, 4-(4-*N*,*N*-dimethylphenyl)-2,6-diphenyl-pyrylium perchlorate, and bis (4-dibutylamino-phenyl)squaraine)) were used as indicators. In addition, a Lewis acid (dinuclear complex of rhodium) and pH indicators (malachite green, *m*-cresol purple, br-cresol purple, dimethyl yellow, phenolphthalein, thymol blue, brilliant yellow, and carminic acid) were also included in the array. The array was placed inside a tray containing marinated turkey but not in direct contact with the meat. Then RGB values of photographs of the trays were analyzed on days 0, 3, 10, 17, 24, 31, 38, and 45 post-storage. Moreover, the aging process of the meet was also studied, and according to the results, the samples were classified into three groups: (i) from day 3 to 24 the samples were classified as “fresh”; (ii) from day 31 to 38, the meat was classified as “not fresh,” and the spoilage process started; (iii) on day 45, the meat was classified as “not edible” because of the high presence of bacteria and other microorganisms. Finally, the colorimetric array response was studied by hierarchical clustering analysis (HCA). The application of this model to the color changes obtained, allowed classifying the samples in concordance with the microbiological data. Color differences from the chromogenic array were employed to create PLS models for aerobic mesophilic bacteria count, sensory score, and storage time with quite good results. 

In another example, fresh pork sausage spoilage was monitored by using a chromogenic array as optoelectronic nose [31]. Freshness over time was also assessed via microbial counts and sensory analyses. The appearance and odor were suitable up to day 24, whereas aerobic counts were detected from day 38. Color differences were analyzed by PCA and partial least squares (PLS). According to the PCA, the array was able to monitor the sausage spoilage process and to distinguish between each sampled day. Moreover, the PLS statistical analysis of the chromogenic data displayed a linear correlation between the predicted and measured values of storage days, mesophilic bacteria, and the sensory score.

Apart from meat aging, optoelectronic noses are also useful to detect food frauds. In this sense, a chromogenic array for the classification of five kinds of cheese (cheddar and four kinds of blue cheese; i.e., Roquefort, Blue Stilton, blue cheese with leaves, and blue cheese spread) was developed [32]. The array was composed of five sensing materials based on the incorporation of pH indicators into MCM-41 and alumina materials as optoelectronic nose. Cheese samples were positioned together with an array in a closed package at a constant temperature for different times (from 0.5 to 8.5 h after packaging). The observations indicate that more volatile substances in cheese reached relatively high concentrations in short times, whereas the concentrations of less volatile compounds increased substantially during the ensuing hours. Then, partial least square discriminant analysis (PLS-DA) was applied to the color changes results, in order to discriminate five cheeses with only five sensing materials. In a first step, the model was applied after only 0.5 h of exposure, and the results indicated a correlation of only 67%. In order to improve those results and to ensure user’s confidence in the above-mentioned technology, data measured at 0.5, 1.5, and 3.5 h were incorporated into the model. The correlation in the classification of cheese improved, in this case, from 67% at 30 min to 100% at 5.5 and 8.5 h. Finally, the same PLS-DA was applied to a lower number of classes. In this case, only three classes of cheese—Roquefort, cheddar, and the remaining blue cheeses—were used after only 30 min of exposure. With this procedure, all three classes of cheese correlated at 100%. 

## 5. Systems Based on Gated Materials

Gated materials are systems based on the use of a porous support that contains “molecular gates.” The porous support is loaded with a certain cargo and then capped by attaching to the external surface a “molecular gate” (or gatekeeper or nanovalve) that inhibits cargo release. Those systems are usually designed to have “zero” release until external stimuli are present. The stimuli can open the gate and allow cargo release. Several porous gated materials able to payload delivery in the presence of physical, chemical, and biochemical stimuli have been reported [33,34,35]. Most gated materials have been designed for drug delivery applications. Moreover, we reported some years ago, the potential use of gated materials as sensing systems. The use of gated materials for sensing applications involves the entrapment as cargo of a dye or fluorophore and the design of the cap in such a way that cargo delivery is selectively observed in the presence of a target analyte. As far as a unique analyte is able to induce dye delivery, this simple concept can be used for the tailored design of chromo-fluorogenic probes for a number of applications.

**Figure 7:**
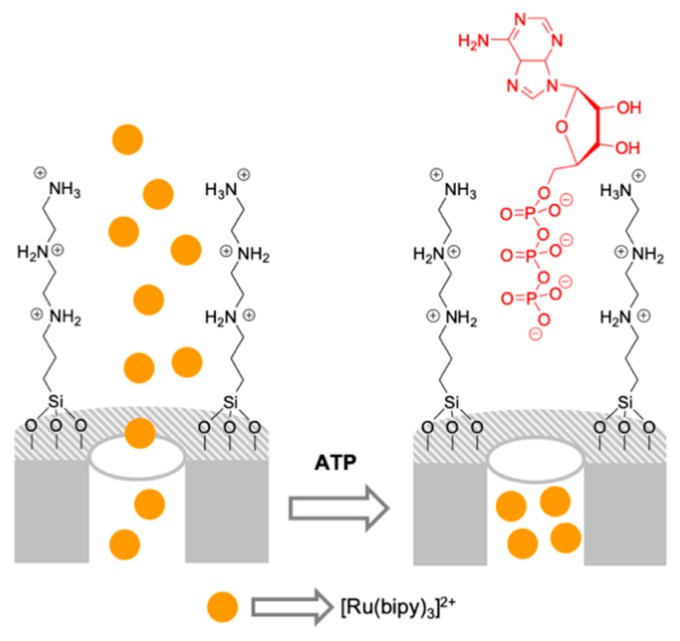
Mesoporous silica nanoparticles capped with polyamines for the selective chromo-fluorogenic detection of ADP and ATP.

The first proof of concept was designed for the detection of nucleotides adenosine diphosphate (ADP) and adenosine triphosphate (ATP) (Figure 7) [36]. In this case, mesoporous silica nanoparticles were functionalized on the external surface with polyamines and the pores were loaded with the dye tris (2,2′-bipyridyl)ruthenium(II) chloride. In the presence of the anions fluoride, chloride, bromide, iodide, nitrate, phosphate, sulfate, acetate, and carbonate, the system remained open and dye delivery was observed. In contrast, in the presence of ADP and ATP nucleotides, no dye release was observed. This was attributed to a capping effect due to a strong electrostatic and hydrogen bonding interaction of the bulky anions ADP and ATP with the anchored protonated amines that inhibited cargo delivery. The extent of this coordination was enough at neutral pH to block the pores, and the strength of the interaction was enhanced by the preorganization effect that arose from grafting the polyamine moieties onto the inorganic support. 

### 5.1. Gated Materias Capped with Biomolecules 

**Figure 8:**
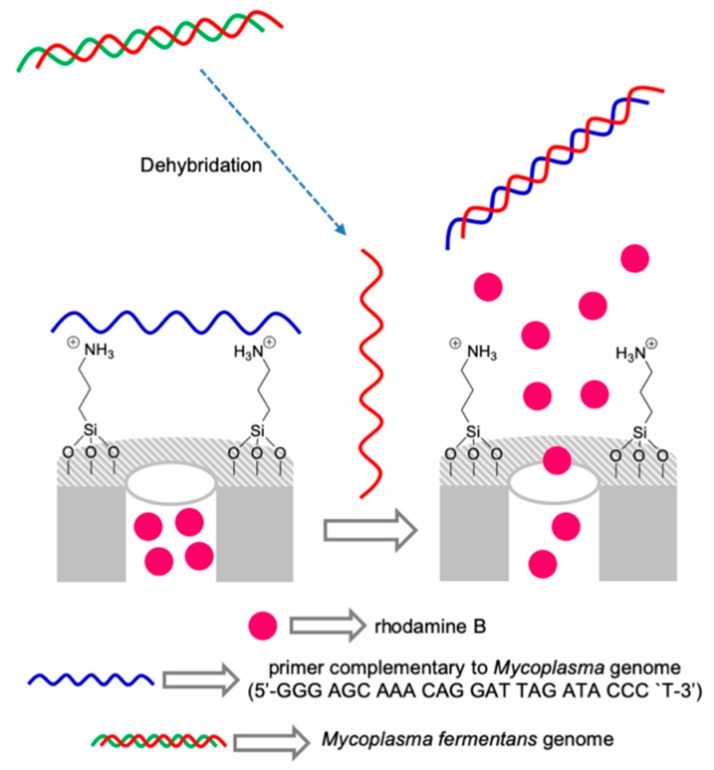
Scheme of a nanogated material capped with a fragment of the 16S ribosomal RNA subunit selective to *Mycoplasma fermentans*. Rhodamine B release was observed in the presence of genomic *Mycoplasma fermentans* DNA.

Based on the concept of using capped materials for the design of chromo-fluorogenic probes, it is possible to design gated nanodevices using biomolecules for sensing applications. A first example using oligonucleotides as capping units was reported in 2010 [37]. In that system the mesoporous material is functionalized with amines, loaded with a dye and capped with selected oligonucleotides. Amines at neutral pH are protonated and can give strong interactions with negatively charged oligonucleotides. It was demonstrated that in the presence of the complementary oligonucleotide a hybridization reaction occurs that results in pore uncapping and dye delivery. In further research, this concept was applied for the detection of DNA of certain microorganism (including pathogens). For instance, an oligonucleotide-capped mesoporous nanomaterial was designed for the detection of *Mycoplasma fermentans*, a prokaryotic microorganism that is a parasite to animals and plants and is considered one of the most common cell-culture contaminants (Figure 8). The oligonucleotide was electrostatically anchored to amine-functionalized mesoporous silica loaded the dye rhodamine B [38]. The capping oligonucleotide corresponded to a fragment of the 16S ribosomal RNA subunit that was selective to *Mycoplasma fermentans*. In the presence of the DNA of this bacterium, a hybridization between the ssDNA from *Mycoplasma* and the capping oligonucleotide occurred resulting in dye release. In contrast, in the absence of *Mycoplasma* genomic DNA or the presence of genomic DNA from other pathogens, such as *Legionella pneumophilia* or *Candida albicans*, negligible dye delivery was observed. A limit of detection of 50 DNA copies·µL^−1^ was achieved. Moreover, the system was used for the detection of *Mycoplasma fermentans* in cell culture media contaminated with this bacterium. The results were compared with PCR method, obtaining comparable results in terms of selectivity and sensitivity. Closely related materials but using nanoporous anodic alumina instead of mesoporous silica were recently developed for the detection *Candida albicans* [39] and *Mycoplasma fermentans* [40].

A similar system was developed for the detection of the α-thrombin, a coagulation protein involved in thrombosis and homeostasis processes, in plasma and human serum with good sensitivity [41]. For this purpose, a specific thrombin-binding aptamer was attached electrostatically to the external surface of mesoporous nanoparticles previously loaded with rhodamine B dye and functionalized with 3-aminopropyltriethoxysilane. In the presence of α-thrombin, there was a displacement of the aptamer to bind the α-thrombin, which resulted in cargo delivery. The limit of detection was evaluated in PBS and commercial human serum, with sensitivity values of 2 and 4 nM resulting, respectively. On the other hand, it was demonstrated that the system was selective and had negligible responses in the presence of other proteins such as ovalbumin (OVA) and bovine serum albumin (BSA).

The same approach, i.e., the use of aptamers as caps, was employed for the detection of small molecules such as cocaine or bisphenol A. For cocaine detection, a combination of surface enhanced Raman scattering (SERS) and gated materials was used [42]. Crystal violet (CV) was selected as cargo and as a SERS reporter. MSNs were externally functionalized with a small oligonucleotide chain. CV was loaded in the pores and the nanoparticles capped with a cocaine aptamer. In the presence of cocaine, the aptamer is displaced from the surface of the nanoparticles into the solution (to bind cocaine), allowing CV release. In a further step, CV reacted with gold nanotriangles that were added to the solution, resulting in a remarkable SERS signal. A LOD of 10 nM for cocaine was determined. It was additionally found that the system was selective, as other drugs (i.e., morphine, methadone, and heroine) induced no SERS signal. The same approach was also applied for the detection of *Mycoplasma fermentans* genomic DNA, in this case using an oligonucleotide selective for *Mycoplasma fermentans* (vide ante). In this case, a LOD of 30 DNA genomic copies·µL^−1^ and good selectivity were observed.

Another example of aptasensor based in capped MSNs was reported for the detection of the carcinogenic molecule Bisphenol A (BPA) [43]. In this case MSNs were loaded with rhodamine B, and then, (3-isocyanatopropyl) triethoxysilane was grafted to the outer surface. A short DNA sequence was anchored to the surface through urea bond formations, and finally, the BPA aptamer was hybridized with the short DNA sequence. Selective uncapping was observed in the presence of BPA, whereas no cargo release was found in the presence of bisphenol C and bisphenol E. The LOD for BPA was established at 3.5 µM. Tap water samples spiked with different concentrations of BPA were successfully analyzed with the aptasensor.

Other sensory materials using aptamers as caps for the detection of As (III) cation [44], ochratoxin A [45], and cocaine [46], were recently described.

Capped materials using antibodies as caps have also been reported for the preparation of chromo-fluorogenic probes for different chemical species. The advantage of using the high selectivity of antibodies in gated materials was first described in 2009 [47]. In this case, a molecule that has a certain affinity for the antibody is anchored on the external surface of a mesoporous support; the system is additionally loaded with a dye; and finally, capped with the antibody. In the presence of the analyte to which the antibody is selective (i.e., the antigen that in this case was sulfathiazole), the antibody is displaced from the material to the solution with the subsequent delivery of the dye.

**Figure 9:**
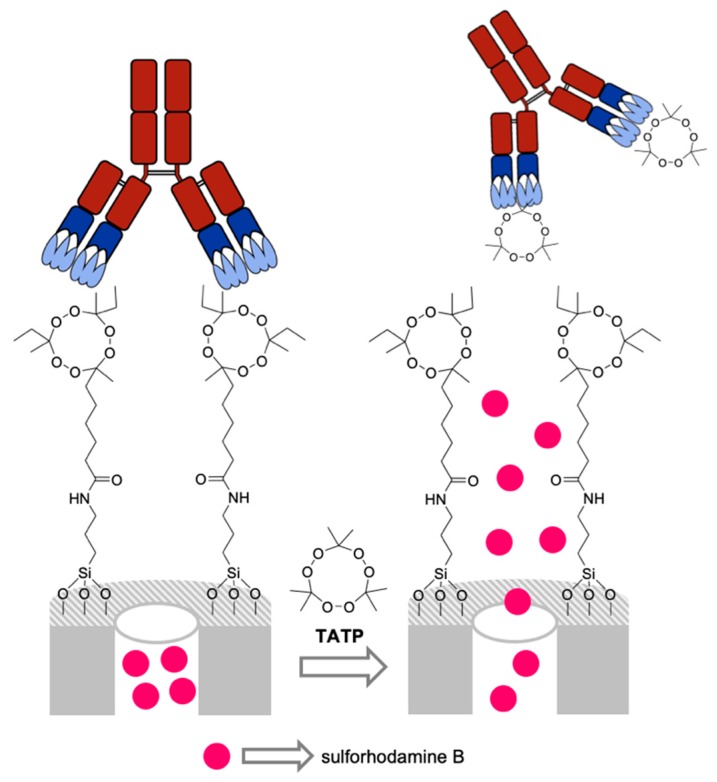
Scheme of antibody-capped, mesoporous silica nanoparticles for TATP detection.

Following this general approach, antibody-capped mesoporous silica nanoparticles were designed for the detection of the popular improvised explosive triacetone triperoxide (TATP) [48]. For this purpose, MSNs were loaded with sulforhodamine B and externally functionalized with a hapten similar to TATP. Finally, a specific TATP antibody was anchored to the surface via hapten affinity capping, avoiding unspecific release (Figure 9). The capped MSNs were tested in solution in the presence and absence of TATP, showing a remarkable dye release in the presence of the explosive. Good selectivity and a LOD as low as 5 ppm for TATP were determined. Once MSNs were optimized, the system was transferred to high-flow nitrocellulose membranes suspending 0.5 µL of the sensing material on the strip surface. The solution with and without TATP raised the recognition zone by capillarity, and then, fluorescence emission was recorded at 625 nm (λ_ex_ 520 nm) with a flow assay reader at the detection zone (strip top). The LOD for the flow assay system was 15 ppb. The same approach, using antibody as capping unit, was used for the preparation of a sensory material to selectively detect steroid finasteride [49].

Capped materials were also designed for the detection of the acetylcholinesterase (AChE) inhibitors diisopropylfluorophosphate (DFP) and neostigmine [50]. MSNs were first loaded with rhodamine B and the external surface was functionalized with pyridostigmine and neostigmine using the corresponding compounds containing ethoxysilane moieties. Finally, the MSNs were capped with AChE via interaction with the pyridostigmine or neostigmine. The probes were suspended in Tris buffer (neutral pH). Both materials were uncapped in the presence of neostigmine and DFP, inducing dye release. However, a better sensitivity was found for the material covered with pyridostigmine with LODs of 0.11 and 3 µM for neostigmine and DFP respectively. Moreover, from selectivity studies it was found that neostigmine, pyridostigmine, DFP, and paraoxon induced the most remarkable rhodamine B release from both materials. Acetylcholine and acetylthiocholine were able to induce moderate rhodamine B release. As a general trend, the fluorogenic response in terms of selectivity was slightly better for the material covered with pyridostigmine. This fact is directly related to the strong coordinating ability of neostigmine with AChE compared with pyridostigmine.

### 5.2. Other Gate-Based Nanosensors

This section includes examples of gated materials as sensors that do not use biomolecules. An example can be found in a reaction between a colorless squaraine-thiol derivative and methylmercury which triggers dye release [51]. Squaraines are known to suffer a nucleophilic attack by thiols forming a colorless covalently linked adduct that can be reversed in presence of Hg^2+^ and methylmercury. The squaraine-thiol adduct is big enough to block the pores of a mesoporous material when grafted onto the external surface. To prepare the sensory nanodevice, mesoporous silica microparticles were prepared and loaded with safranin O. Then, the external surface was functionalized with mercaptopropyl moieties and the pores capped upon addition of a squaraine derivative. In the presence of methylmercury, a marked safranin O delivery was observed due to the reaction of methylmercury with the capping molecule that released the anchored squaraine and the entrapped dye. The observed response is selective to methylmercury because a negligible safranin O release was observed in the presence of Na^+^, K^+^, Ca^2+^, Mg^2+^, Cu^2+^, Ni^2+^, Zn^2+^, Ag^+^, Pb^2+^, Cd^2+^, and Au^3+^ cations. Besides, the LOD for the detection of methylmercury using color changes is 0.5 ppm and the use of fluorescence reduces this value to less than 2 ppb. Although the chemical principles were known, this approach does not use the squaraine as a signaling unit; instead, it benefits from the possibility that the number of molecules of safranin O inside the pores is much higher than the number of squaraines in the surface. Upon addition of an atom of methylmercury, the pore opens, releasing numerous molecules of the safranin O dye with the consequent improvement of sensitivity by signal amplification. 

**Figure 10:**
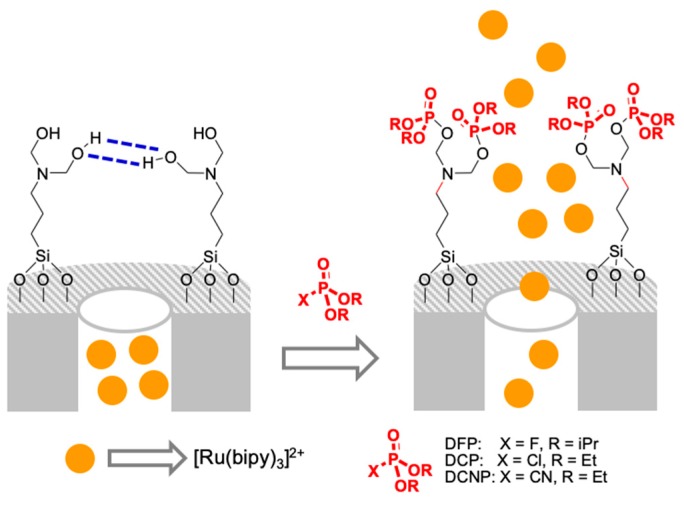
Left: scheme of the MSNs-capped system for the detection of nerve agents.

Silica-based gated materials have also been used for the detection of nerve agent mimics through a pore-opening protocol (Figure 10) [52]. MSNs were first loaded with [Ru(bipy)_3_]Cl_2_, and then, pores were blocked by bis(2-hydroxyethyl)aminopropyltriethoxysilane (HET). Due to the external hydrogen-bonding network formed, dye release was inhibited. In the presence of nerve agent mimics, such as diethyl chlorophosphate (DCP), diisopropyl fluorophosphate (DFP), diethyl cyanophosphonate (DCNP), and to a lesser extent diethyl chlorothiophosphate (DCTP), the electron-deficient phosphorous atoms of these agent mimics reacted with the OH groups of the gated ensemble, forming stable phosphate derivatives. This broke down the hydrogen-bond barrier, resulting in ruthenium dye release. Experiments were undertaken in acetonitrile solutions and LODs of ca. 0.1 ppm were achieved for DCP, DFP, and DCNP.

**Figure 11:**
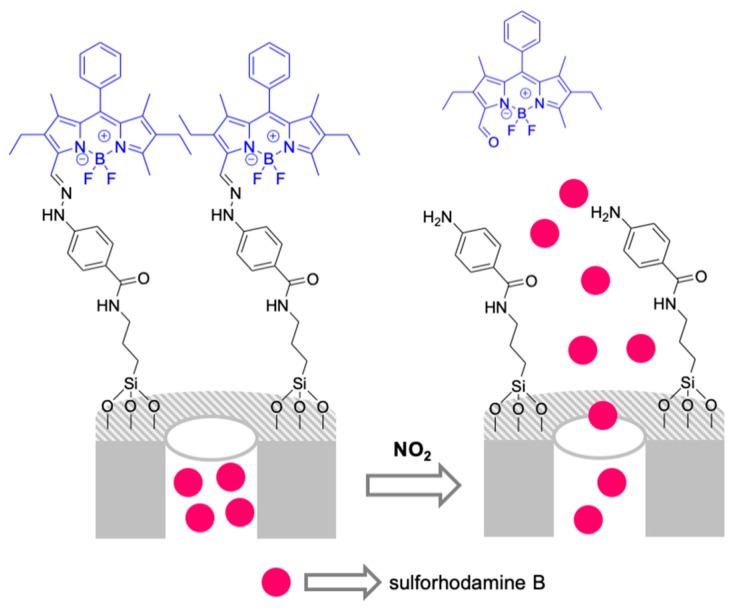
Scheme of the MSNs-capped system for the detection of NO_2_.

A capped system able to uncap in the presence of NO_2_ was reported for the naked-eye detection of this gas in solution (Figure 11) [53]. MSNs were loaded with sulforhodamine B dye and capped with a difluoroboron-dipyrromethene (BODIPY) derivative. The uncapping mechanism was based on NO_2_ oxidative regeneration of carbonyl compounds that resulted in BODIPY cleavage and cargo release. As a particularity, in this system NO_2_ can be detected by monitoring the dye released from the pores or the dye generated during the gate cleavage. In a typical experiment, the solid was suspended in acetonitrile and then NO_2_ gas was bubbled, resulting in a clear color change of the suspension from colorless to magenta due to sulforhodamine B dye release. A LOD of 0.11 ppm for NO_2_ was achieved, which was much lower than the limit established by the European Union for nitrogen dioxide in the air. At the same time, the system was highly selective, and no dye delivery was observed in the presence of other gases, such as NO, CO_2_, H_2_S, and SO_2_, nor were vapors from acetone, hexane, chloroform, acetonitrile, and toluene.

Hydrogen sulfide (HS^−^) was detected using MSNs capped with a coordination complex using a demetallation process. The probe, based on MSNs, was loaded with [Ru(bipy)_3_]Cl_2_, functionalized with a Cu^2+^-triazapyridinophane complex, and capped with the bulky anion hexametaphosphate through electrostatic interactions with the positively charged Cu^2+^ complex [54]. In the presence of HS^−^, the demetallation of Cu^2+^ resulted in dye release. The selectivity of the system to HS^−^ was demonstrated, as no other anions (i.e., F^−^, Cl^−^, Br^−^, I^−^, N_3_^−^ CN^−^, OH^−^, HPO_4_^−^, NO_3_^−^, HCO_3_^−^, SO_4_^2−^, AcO^−^ and citrate), aminoacids (i.e., Ala, His), thiol-containing biomolecules (i.e., Cys, HCy, GSH, Me-Cys), or oxidants (i.e., H_2_O_2_ and S_2_O_8_^2−^) were able to induce cargo release. A LOD as low as 1.85 µM for HS^−^ was determined. The nanoprobe was used to detect HS^−^ in realistic environments, such as tap water (83% to 92% recoveries).

The MSNs system for the controlled release of insulin with the aim of controlling glucose concentration changes in diabetes patients was developed [55]. Expanded-pore nanometric silica particles (pore diameter of 11.8 nm, nanoparticle diameter 426 nm) were loaded with fluorescein-labeled insulin and externally grafted with 1-propyl-1-*H*-benzimidazole groups. Then, capping was achieved by the formation of inclusion complexes between cyclodextrin-modified glucose oxidase (CD-GOx) and previously attached benzimidazole moieties. The resulting nanodevice was tested in simulated blood plasma (neutral pH) in the presence and the absence of 50 nM glucose concentration and other sugars, such as mannose, fructose, galactose, and saccharose. In the presence of glucose, gluconic acid formation by CD-GOx enzymatic activity promoted cargo release due to a pH decrease, benzimidazole protonation and dethreading of the inclusion complexes. Cargo release was determined by monitoring insulin-dye release to the solution. Finally, additional experiments determined an amount of 250–300 µg of the nanodevice was required to reach correct and controlled insulin levels.

MSNs capped with pseudo-rotaxanes have been designed for the selective and sensitive detection of 3,4-methylenedioxymethamphetamine (MDMA, also known as ecstasy) in water [56]. For this purpose, MSNs were loaded with fluorescein, externally functionalized with a naphthalene derivative, and finally, capped via the formation of inclusion complexes between the anchored 1-naphtol subunits from the scaffold and the cyclobis(paraquat-*p*-phenylene)hexafluorophosphate macrocycle ([CBPQT][PF_6_]_4_). Negligible fluorescein release was observed in aqueous suspensions of the nanoparticles at pH 7 in the absence of MDMA, whereas in the presence of the target drug, a remarkable dye release was observed. The uncapping mechanism can be attributed to the preferential coordination of CBPQT^4+^ macrocycle with MDMA rather than with superficial naphtol moieties. Selectivity was demonstrated testing the sensing system in the presence of other common, drugs such as cocaine, heroin, methadone, and morphine, being that all of them were unable to induce fluorescein release. Finally, a LOD of 4.9 mM for MDMA in water was determined.

Finally, in most of the systems reported above, the analyte activates the release of the dye inside the pores (OFF–ON approach). In the next example, the analyte blocks dye release, generating a reduction in the dye that is delivered (ON–OFF approach) [57]. For this purpose, the pores of the MSNs were loaded with [Ru(bipy)_3_]Cl_2_ and the external surface functionalized with imidazolium groups that are known to bind carboxylates. In a typical experiment, the solid was immersed in water solutions containing different carboxylates (CH_3_(CH_2_)_n_COO^−^, (i.e., acetate, butanoate, hexanoate, octanoate, decanoate, and dodecanoate (n = 0, 2, 4, 6, 8, and 10, respectively)) in equal concentrations (10^−3^ M). In the presence of long-chain carboxylates (n ≥ 8), pore blockage was accomplished, and dye delivery was inhibited. This inhibition was only reached with anionic long-chain carboxylates, because once they had coordinated with imidazolium group, a hydrophobic layer was formed, disabling dye delivery. To prove that blocking capacity was not caused by simple interaction with the silica surface, a solid with no imidazole functionalization was prepared and tested in the same conditions, resulting in no inhibition of dye delivery in the presence of carboxylates.

The applications of gated materials in recognition protocols have attracted great attention and systems similar to that described above for the detection of acetylcholine [58], endotoxin [59], miRNA-145 [60], glutathione [61], nitroaromatic explosives [62,63,64,65], borate [66], and glucose [67] were recently described.

### 5.3. Recent Advances on Gated Nanosensors

As an evolution of the porous materials capped with biomolecules, our group has been developing bio-inspired systems for the design of optical probes. At this respect, we reported recently, a probe for urea detection based in a nanodevice that combines an enzyme, as the recognition unit, with a controlled release system in separate parts of the same particle [68]. For the preparation of the sensing nanodevice, Janus gold-mesoporous silica nanoparticles were selected as the inorganic scaffold and the different faces were properly functionalized. Thus, the gold face was functionalized with urease enzyme and the mesoporous silica face was functionalized with amine moieties and then capped with an oligonucleotide labeled with Alexa-Fluor-674 fluorophore. In the presence of urea, a marked emission enhancement was observed, which was ascribed to the recognition of urea by the enzyme on the gold face of the nanodevice, which catalyzed the formation of carbon dioxide and ammonia. This ammonia induced the deprotonation of the amine groups on the silica face with the subsequent release of the labeled oligonucleotide. The optical response of the nanodevice was selective. Thus, among all biomolecules tested (glucose, creatinine, uric acid, and urea), only urea was able to induce the detachment of the labeled oligonucleotide. This simple nanodevice was applied for the detection of urea in real human blood samples and for the identification of adulterated milk. 

In another, more advanced example, gated Janus nanoparticles have recently been used for the development of cooperative systems in which nanoparticles are able to talk each to another [69,70] or in communication systems between nanoparticles and cells [71]. In this sense, we have recently reported that sucrose triggers the communication between yeasts and Janus nanoparticles, inducing the expression of the green fluorescent protein in yeast that can be followed instrumentally. More in depth, Janus gold-mesoporous silica nanoparticles were prepared bearing glucose oxidase enzyme on the gold face, whereas the pores in the silica face were loaded with phleomycin (a DNA-damaging agent which induces RNR3 gene transcription and green fluorescent protein expression in *Saccharomyces cerevisiae* yeast) and capped with a pH-sensitive supramolecular ensemble formed by the inclusion of β-cyclodextrin with a grafted benzimidazol heterocycle. The communication cascade starts upon addition of sucrose which was hydrolyzed by the yeast onto glucose and fructose. Then, glucose was transformed, by the enzyme immobilized onto the gold face of the nanodevice, to gluconic acid. This acidic medium induced benzimidazol protonation and dethreading of the inclusion complex. As a consequence, pores were opened and phleomycin released to the environment. This drug was endocytosed by the yeast and induced the expression of green fluorescent protein. These findings demonstrated the ability of abiotic nanodevices to sense the surrounding environment and detect certain chemicals (in this case glucose) and act accordingly (pore opening and release of a second molecule), resulting overall, in a cell-nanoparticle biunivocal communication. These or related nanodevices, capable of communicating with cells, can have impact on the preparation of new strategies for new sensors and diagnosis protocols.

## 6. Conclusions

We have offered here, an overview of the evolution of the silica-based, colorimetric nanosensors during the last decades through relevant research developed in our group. Colorimetric sensors have benefited from advances in the areas of supramolecular chemistry and nanomaterials, yielding a new generation of systems with the potential of offering improved sensitivity and/or selectivity. Following the evolution of this area, a clear increase in the complexity of the systems from simple grafting of responsive molecules to a new generation of materials containing binding pockets or molecular gates in which the silica nanoscopic properties are key elements of the sensor can be seen. Moreover, we believe that some of the strategies developed in this review can be transferred to other materials, and at the same time, the development of silica-based colorimetric sensors can benefit from the combination of the silica support with many other nanostructures. In this sense, above we have described an example of the integration of silica with gold nanotriangles, in which the substance released from silica modifies the properties of the gold nanostructure.

In parallel, in recent years, there has been a revolution in the collection and treatment of color changes through digitalization and smartphones, with the ability to multiply the applications in the near future. However, in most cases the systems are in the form of discrete dust or particles, making the integration with the equipment difficult, along with its automation, and finally, its application in the real world. This is a key element that will be addressed by researchers in the future. Along with automation issues, we can expect important developments in the use of this class of systems not only for the analysis of samples but for the analysis of certain biomolecules within in vivo models. By bearing all these facts in mind, new advances in the use of nanosilica for the development of colorimetric sensors are anticipated in the near future.
